# An unexpected role for leucyl aminopeptidase in UV tolerance revealed by a genome-wide fitness assessment in a model cyanobacterium

**DOI:** 10.1073/pnas.2211789119

**Published:** 2022-11-02

**Authors:** Elliot L. Weiss, Mingxu Fang, Arnaud Taton, Richard Szubin, Bernhard Ø. Palsson, B. Greg Mitchell, Susan S. Golden

**Affiliations:** ^a^Integrative Oceanography Division, Scripps Institution of Oceanography, University of California San Diego, La Jolla, CA 92093;; ^b^Department of Molecular Biology, University of California San Diego, La Jolla, CA 92093;; ^c^Center for Circadian Biology, University of California San Diego, La Jolla, CA 92093;; ^d^Bioengineering Department, University of California San Diego, La Jolla, CA 92093

**Keywords:** UV radiation, cyanobacteria, fitness, leucyl aminopeptidase, RB-TnSeq

## Abstract

UV radiation (UVR) has significant physiological effects on organisms living at or near the Earth’s surface, yet the full suite of genes required for fitness of a photosynthetic organism in a UVR-rich environment remains unknown. This study reports a genome-wide fitness assessment of the genes that affect UVR tolerance under environmentally relevant UVR dosages in the model cyanobacterium *Synechococcus elongatus* PCC 7942. Our results highlight the importance of specific genes that encode proteins involved in DNA repair, glutathione synthesis, and the assembly and maintenance of photosystem II, as well as genes that encode hypothetical proteins and others without an obvious connection to canonical methods of UVR tolerance. Disruption of a gene that encodes a leucyl aminopeptidase (LAP) conferred the greatest UVR-specific decrease in fitness. Enzymatic assays demonstrated a strong pH-dependent affinity of the LAP for the dipeptide cysteinyl-glycine, suggesting an involvement in glutathione catabolism as a function of night-time cytosolic pH level. A low differential expression of the LAP gene under acute UVR exposure suggests that its relative importance would be overlooked in transcript-dependent screens. Subsequent experiments revealed a similar UVR-sensitivity phenotype in LAP knockouts of other organisms, indicating conservation of the functional role of LAPs in UVR tolerance.

## Significance

Cyanobacteria account for ∼25% of global primary production and dominate vast regions of the ocean, as well as soil crusts. While they rely upon light availability for photosynthesis, they are simultaneously exposed to UV radiation (UVR). Damage induced by UVR decreases photosynthetic rates, suggesting that previous calculations of global primary production may be overestimated. Despite the strong impact of UVR on phytoplankton, there has not yet been a genome-wide fitness assessment to identify genes that are important for UVR tolerance in a photosynthetic organism under environmentally relevant UVR dosages. We report genes that are most critical for UVR tolerance in a cyanobacterium and the function of a conserved leucyl aminopeptidase whose disruption results in loss of fitness under UVR.

UV radiation (UVR, 300 to 400 nm) is the most photochemically reactive waveband of the solar spectrum and can have deleterious effects on the physiology of organisms that live on Earth’s surface or in the near-surface region of lakes and oceans. While photosynthetic organisms primarily rely on photosynthetically available radiation (PAR, 400 to 700 nm) and near-infrared radiation for photosynthesis, they are not immune to the harmful effects of UVR that are concurrent with PAR. These effects can occur at biologically relevant levels in the water column to depths of up to 20 m (1, 2). In the era of anthropogenically induced climate change, defined by changes to ice melt, aerosol concentrations, cloud cover, the ozone layer, and variations in water-column mixed layer depths, phytoplankton production and community structure will respond directly to related changes in UVR dosage, although these changes remain difficult to predict (3). Natural populations of phytoplankton will respond differently to changes in UVR stress; for example, many large-celled microbes can efficiently utilize sunscreen molecules as a photoprotective mechanism and are often less affected by high light irradiances and UVB (290 to 320 nm), while picoplankton typically experience more damage (4, 5).

Types of damage induced by UVR in phytoplankton have long been understood, such as the formation of reactive oxygen species (ROS), direct damage to DNA and proteins, and the inhibition of photosynthesis (6, 7). The general mechanisms of tolerance are thus considered to be DNA repair, protein repair and recycling, and the synthesis of antioxidants, UV-absorbing sunscreens, and photoprotective molecules, as well as avoidance of UVR via motility and mat formation (7). Most studies on UVR tolerance in phytoplankton have targeted specific processes in these categories that were previously shown to confer resistance, using transcript-dependent methodologies such as RNA Sequencing (RNA-Seq) to detect UVR-regulated genes as a proxy for gene relevance. While useful, screens of this nature do not provide direct measures of gene-specific fitness impacts. Additionally, there is a necessity to provide realistic UVR spectra in these studies, as previous research has demonstrated that the harmful effects of UVR on phytoplankton are highly wavelength dependent; wavelengths in the UVB region inhibit photosynthetic activity exponentially more than longer wavelengths in the UVA region, and direct damage to DNA and proteins occur as a result of UVB exposure (7, 8). A dearth of genome-wide fitness screens for UVR tolerance in phytoplankton, under realistic conditions, suggests that mechanisms and genes important for UVR tolerance remain to be discovered.

Random barcode transposon site sequencing (RB-TnSeq) is a methodology that has proven to be particularly useful for elucidating genes that are most important to sustain a variety of conditions and processes in the model cyanobacterium *Synechococcus elongatus* PCC 7942 (9–12). In this study we leveraged RB-TnSeq to elucidate the gene set that is most critical in *S. elongatus* for tolerating UVR stress under a light cycle that resembles natural UVR dosage and short-wavelength spectral quality. Our data highlight the importance of a leucyl aminopeptidase with cysteinyl-glycinase activity that was previously overlooked and support the involvement of many canonical processes in UVR tolerance.

## Results

### Physiological Response and Transcriptomic Landscape of Wild-Type *S. elongatus* Grown under a Light:Dark Cycle with Three UVR Dosages.

**Fig. 1.**
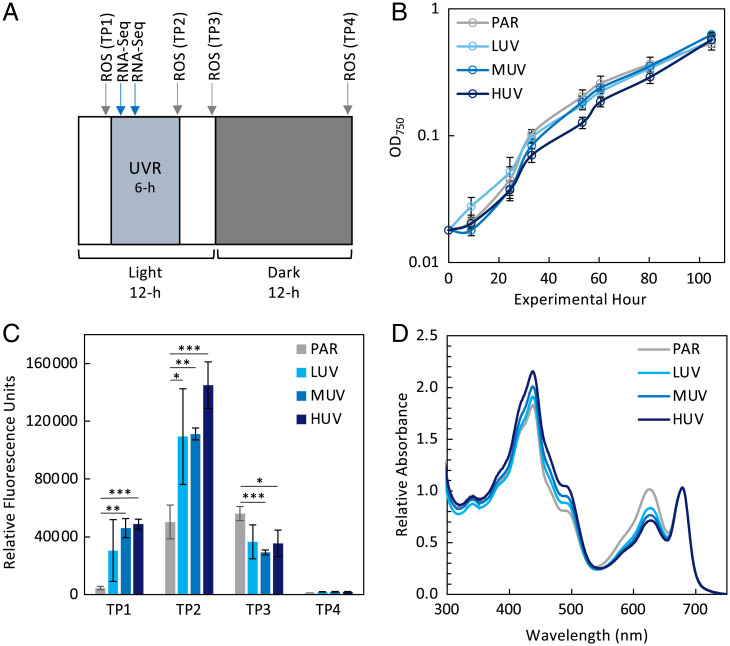
WT growth and physiological response to PAR-only, LUV, MUV, and HUV conditions. (*A*) Schematic of 12-h light:12-h dark cycle and 6-h UVR period, including ROS sampling timepoints (TP1 to 4), and 15 min and 2 h RNA-Seq sampling points, which were collected after three light:dark cycles. (*B*) OD_750_ measured over 105 h. (*C*) ROS measured in the whole cell fraction by H_2_DCFDA fluorescence following three light:dark cycles. ROS measurements were taken at each timepoint illustrated in *A*, including the end of the daily first PAR-only period (TP1), UV period (TP2), second PAR-only period (TP3), and dark period (TP4). (*D*) Whole cell absorption spectra of each UVR condition after 4 d of growth normalized to 675 nm. Data points are mean (SD); *n* = 3.

A suite of experiments was conducted to assess how *S. elongatus* is affected by three environmentally relevant UVR dosages. Wild-type (WT) cultures of *S. elongatus* grown to exponential phase and transferred to quartz photobioreactors were exposed to a 12-h light:12-h dark cycle with the addition of UVR for the central 6 h of the light period at one of three dosages levels: low UVR (LUV), medium UVR (MUV), or high UVR (HUV), or the PAR-only control (Fig. 1*A*). Growth rates were equivalent among the LUV, MUV, and PAR-only conditions throughout the experimental period. However, cells in the HUV condition grew more slowly than the PAR-only control during an initial 53-h phase, after which all growth rates were equivalent (experimental h 53 to 104) (Fig. 1*B*). ROS measured in cultures over the course of the light cycle illustrated a distinct pattern of ROS formation and dissipation (Fig. 1*C*). At the end of the 6-h UVR exposure period, ROS was higher in all UVR samples relative to the PAR-only control (*P* value LUV <0.05; MUV <0.01; and HUV <0.001). At the end of the following 6 h of white light, ROS levels in the UVR treatments dropped below the PAR-only treatment. No ROS was detected the next morning following 12 h of darkness, implying that cells were able to fully scavenge all ROS and/or the ROS had fully reacted with cellular targets. Surprisingly, after the 6-h white-light period at virtual dawn, there was significantly greater ROS detected in the UVR samples, despite no UVR exposure for this period. It is possible that an accumulation of damaged DNA and proteins from the UVR exposure of the prior light cycle stimulated enhanced photosynthetic activity, resulting in elevated ROS. Whole cell absorption spectra normalized to 675 nm showed pronounced variations in the ratio of the 630-nm to the 675-nm peak, indicating greater absorption by phycobiliproteins (PBPs) in PAR-only relative to UVR treatments and suggesting either the down-regulation of PBPs or destruction of PBPs under UVR, which is consistent with prior studies if we assume that there was little change in the chlorophyll concentration (Fig. 1*D*) (13).

**Fig. 2.**
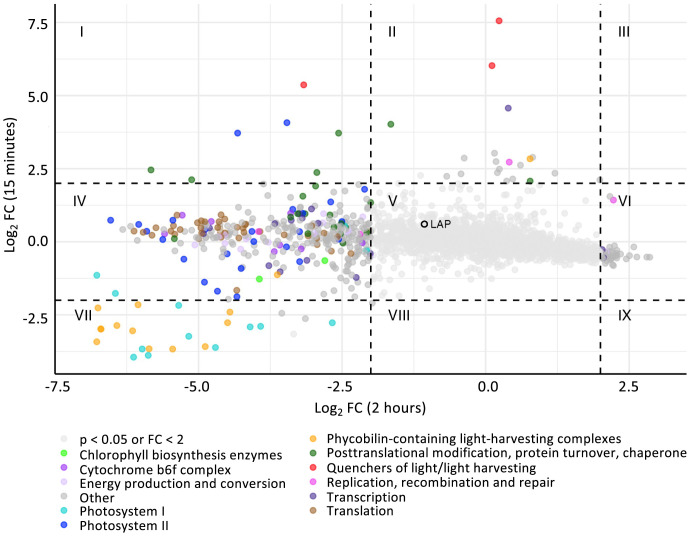
Differential expression of genes in the WT strain after 15 min (*y* axis) or 2 h (*x* axis) of HUV exposure relative to PAR-only. Genes are color coded by select functional families. Dotted lines indicate the ±2 log_2_ fold change thresholds, with roman numerals indicating genes that are: *I*) up-regulated at 15 min and down-regulated at 2 h, *II*) up-regulated at 15 min only, *III*) up-regulated at 15 min and 2 h, *IV*) down-regulated at 2 h only, *V*) without strong differential expression, *VI*) up-regulated at 2 h only, *VII*) down-regulated at both 15 min and 2 h, *VIII*) down-regulated only at 15 min, and *IX*) down-regulated at 15 min and up-regulated at 2 h of UVR exposure. The leucyl aminopeptidase gene *Synpcc7942_1190* is highlighted in section *V*.

Differential expression of genes during the UVR period was analyzed 15 min and 2 h after the onset of the UVR period following three light:dark cycles under the HUV condition using RNA-Seq. After 15 min of UVR exposure, 28 genes were up-regulated (log_2_ fold change >2; adjusted *P* value <0.05), while 18 were down-regulated (Fig. 2). Transcripts strongly down-regulated upon 15 min of UVR exposure primarily encode proteins related to photosystem I subunits and phycobiliprotein-containing light-harvesting complexes. Transcripts most up-regulated upon 15 min of UVR exposure encode proteins involved in light quenching, the synthesis of photosystem II D1 protein isomers PsbA2/PsbA3; protein turnover; carotenoid cycling; transcription; and replication, recombination, and repair. After 2 h of UVR exposure, 41 genes were differentially up-regulated, while 377 were down-regulated. Strongly down-regulated genes relate to photosystems I and II, energy conversion, phycobiliprotein-containing light harvesting complexes, and translation. Of the transcripts belonging to the “translation” Clusters of Orthologous Groups (COG) (14) functional group, those showing strong down-regulation included the *rpl*, *rpm*, and *rps* gene families. A complete list of the differential expression of transcripts is provided in Dataset S1.

### Assessment of Genome-Wide Fitness under UVR Using RB-TnSeq.

A fresh culture of the RB-TnSeq mutant library of *S. elongatus* PCC 7942, recovered from a frozen archive following a standardized protocol (9), was entrained in a 12-h light:12-h dark cycle for 48 h and inoculated into quartz tubes; cultures were incubated with or without the addition of a 6-h LUV, MUV, or HUV exposure period to a 12-h light:12-h dark regimen. The cultures were diluted to an OD_750_ of 0.05 halfway through the experimental period to minimize the attenuation of incident light. After five to six generations of growth the library was harvested and the barcodes were sequenced (9). Based on the relative number of barcode sequence reads per gene under each UVR condition to the PAR-only condition, we were able to quantify the relative fitness contribution of each gene under each UVR dosage level (Dataset S2).

**Fig. 3.**
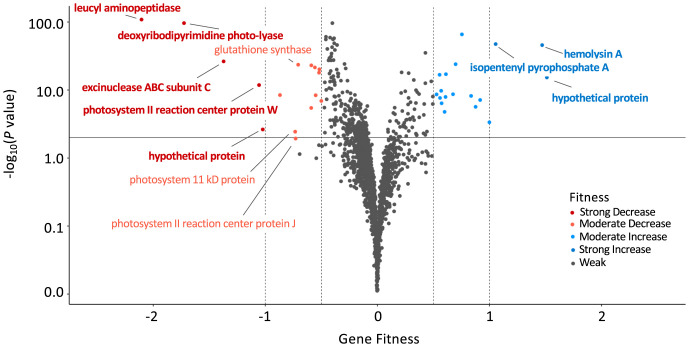
A volcano plot highlighting genes whose loss have moderate to strong fitness effects under HUV. Colors indicate whether loss of a gene causes an estimated fitness decrease (red) or increase (blue). A horizontal line indicates the *P* value cutoff threshold, and dotted vertical lines indicate the cutoff thresholds for moderate and strong fitness effects (−1, −0.5, 0.5, and 1). Bold text highlights genes with the strongest fitness effects (< −1 or >1).

*S. elongatus* PCC 7942 has 2,723 genes in its 2.7-Mbp genome, of which 718 were previously determined to be essential (9); thus, those mutants are absent from the library and were not sampled in this assay. The essentiality status of all genes that were differentially expressed after acute UVR exposure is available in Dataset S1. Our experiment revealed that 5 of the nonessential genes, when disrupted, conferred a strong decrease in fitness specifically under the HUV condition relative to the PAR-only condition (log_2_ fold < −1.0; false discovery rate [FDR] adjusted *P* value <0.05), and mutations in 14 additional genes led to a moderate decrease in fitness (log_2_ fold < −0.5; FDR adjusted *P* value <0.05) (Fig. 3). The top 5 mutant loci that strongly decreased fitness are *Synpcc7942_1190*, *Synpcc7942_0112*, *Synpcc7942_1945*, *Synpcc7942_1679*, and *Synpcc7942_1616*. These genes are currently annotated as encoding a leucyl aminopeptidase (LAP), a deoxyribodipryrimidine photolyase, an excinuclease ABC subunit C, a photosystem II reaction center W protein, and a hypothetical protein, respectively. A strong increase in fitness was calculated only for mutants with insertions in 3 genes: *Synpcc7942_1628* (hypothetical protein), *Synpcc7942_0319* (hemolysin A), and *Synpcc7942_1933* (isopentenyl pyrophosphate isomerase). However, closer examination of the data revealed that the positive values in these cases resulted from exceptionally poor representation of these barcodes in the PAR-only sample rather than enhanced growth in PAR + HUV, indicating an amelioration of the mutant defect under UVR. The same was true for other mutant loci that were identified as moderately improving fitness.

Four of the top five genes revealed by the RB-TnSeq screen to be important under HUV exhibited a fitness response that is linearly correlated with the UVR dosage incident on the mutant pools. For example, mutants defective for *Synpcc7942_1190* had a decrease in log_2_ fold fitness values under HUV, MUV, and LUV of −2.1, −1.6, −0.9 (*P* < 0.01, and FDR <0.01; *t* test), respectively. Only *Synpcc7942_1616* exhibited a dosage-independent response (HUV: −1.0, MUV: −1.1, and LUV: −0.8), suggesting that the mechanism of UVR tolerance conferred by this gene is saturated even at lower dosages of UVR.

The genes whose loss confers moderate to strong fitness decreases were classified and subsequently binned into functional groupings based on merged COG and cyanobacterial clusters of orthologous groups of proteins (CyoG) (15) databases. One is classified as being involved in coenzyme transport and metabolism (*gshB*); one in energy production and conversion (*Synpcc7942_0567*); one in nucleotide transport and metabolism (*codA*); three in photosystem II (*psb27*, *psb28-1*, and *psbJ*); one in posttranslational modification and protein turnover (*ppiB*); three in replication, recombination, and repair (*phr*, *uvrC*, and *ssb*); one in translation (*miaA*); and eight were unclassified by the database (*Synpcc7942_1190*, *Synpcc7942_1616*, *Synpcc7942_0037*, *Synpcc7942_2194*, *Synpcc7942_B2641*, *Synpcc7942_1812*, *Synpcc7942_B2643*, and *Synpcc7942_1511*).

### LAP Knockout Phenotype Verification and Complementation.

**Fig. 4.**
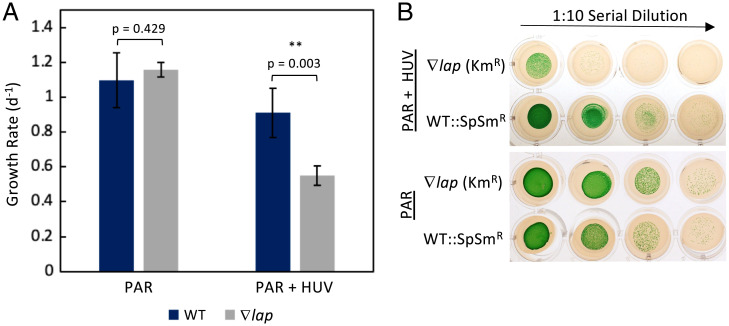
Results of a coculture growth competition between a *lap*-deficient mutant and the WT strain. (*A*) Growth rates under the PAR-only and PAR + UVR condition. (*B*) CFUs from spots of the coculture under PAR and PAR + UVR conditions after 7 d of growth, then serially diluted and plated on selective media. The images shown are representative of those obtained in three independent experiments. Data points are mean (SD); *n* = 3.

**Fig. 5.**
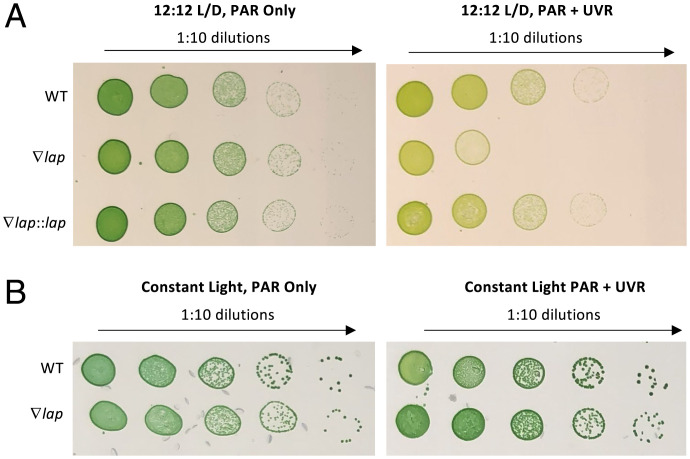
Spot tests reveal that the *lap* mutant can be complemented, and that the sensitivity to UVR is enhanced in a light:dark cycle relative to constant light. The 5-µL spots, serially diluted from a starting OD_750_ of 0.25, of the WT and mutant strains of *S. elongatus* grown under PAR-only or PAR and HUV. (*A*) Cells grown under 12-h light:12-h dark cycle. (*B*) Cells grown under constant light, with UVR present for 6 h per day. The images shown are representative of those obtained in three independent experiments.

The greatest decrease in fitness observed under our HUV condition was conferred by mutations in *Synpcc7942_1190*, a homolog of the *Escherichia coli pepA* gene (UniProt accession no. P68767), called *lap* in *Synechocystis* sp. PCC 6803 (16), which encodes a putative leucyl aminopeptidase. We assessed the phenotype of a knockout mutant of *S. elongatus Synpcc7942_1190*, hereafter called *lap*, and performed kinetic assays of the LAP protein. To further examine the UVR-sensitive phenotype identified by the RB-TnSeq screen, a fully segregated insertion mutant of *lap* with a kanamycin-resistance marker was generated (∇*lap*) and grown in coculture with a WT strain containing a spectinomycin–streptomycin marker integrated at genomic neutral site 2 (WT::SpSm^R^) for 8 d under both the PAR-only control and HUV conditions used in the library screen (17). Samples were collected daily and plated on both kanamycin and spectinomycin–streptomycin selective media, and colony forming units (CFUs) were used to calculate growth rates (Fig. 4). There was no statistical difference in growth rate under the PAR-only condition; however, growth of the ∇*lap* mutant was significantly inhibited under HUV relative to the WT (*P* < 0.05; Fig. 4 *A* and *B*). To ensure that the phenotype of the ∇*lap* mutant is not the result of a second-site mutation elsewhere in the genome, the ∇*lap* mutant was complemented with a segment of DNA extending from 243 bp upstream to 71 bp downstream of *SynPCC7942_1190* inserted into neutral site 1 of the genome (∇*lap*::*lap*) (17). When spotted and grown on BG-11 agar, the WT phenotype was restored in the ∇*lap*::*lap* strain (Fig. 5*A*). To assess whether the ∇*lap* mutant experienced enhanced sensitivity due to the dark period of the light–dark cycle in combination with prior UVR exposure, an identical experiment was conducted between the ∇*lap* mutant and the WT under constant light, with the UVR lamp active during the same 6-h exposure period as in previous studies. While the CFU circumference of the ∇*lap* mutant was reduced relative to the WT, the UV-sensitive phenotype, as assessed by reduced CFU viability in the 12-h light:12-h dark + UVR condition, was alleviated. This result indicates that the lethality of a *lap* insertional mutant relative to the WT when exposed to UVR is enhanced under the ecologically relevant condition of a subsequent period of darkness (Fig. 5*B*).

### Recombinant Expression and Purification of SynPCC7942_1190 in *E. coli* and Characterization of Enzymatic Properties.

**Fig. 6.**
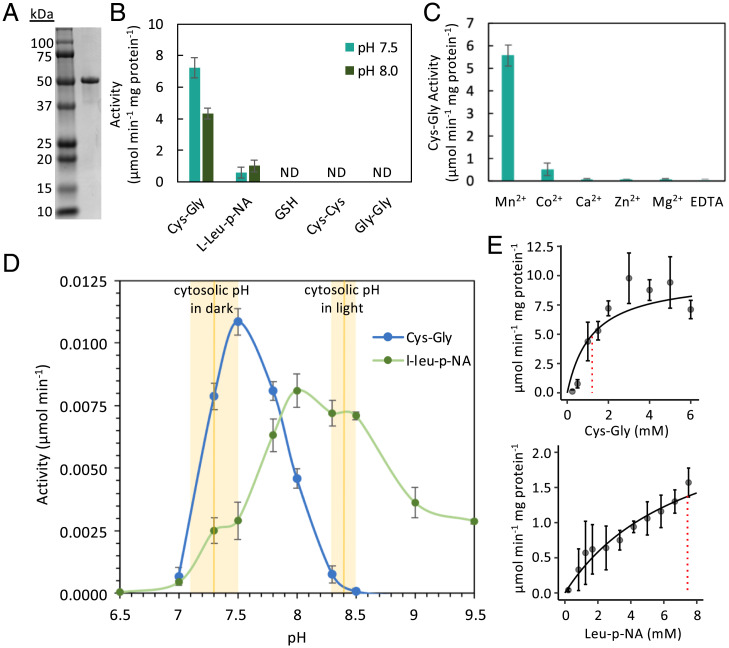
Kinetic assays of recombinant Synpcc7942_1190 Lap protein reveal preferential affinity for cysteinyl-glycine. (*A*) SDS-PAGE gel of the 51-kDa recombinant Synpcc7942_1190 Lap protein. (*B*) Activity of the recombinant protein against various substrates, at pH 7.5 and 8.0. (*C*) Activity of the recombinant protein against cysteinyl-glycine in the presence of metal cofactors. (*D*) Activity of the recombinant protein against cysteinyl-glycine and L-leucine-p-nitroanilide over a pH gradient. Average intracellular pH levels of *S. elongatus* in the dark and light are overlaid, per Mangan et al. (31). (*E*) Samples containing 5 µg/mL of the recombinant protein were assayed for cysteinyl-glycine or L-leucine-p-nitroanilide at different substrate concentrations at pH 7.5. *K*_m_ values are overlaid vertically as red dotted lines. Data points are mean (SD); *n* = 3.

SynPCC7942_1190 is predicted to be of the M17 family of proteases and function as an LAP; however, prior research has shown that some LAPs have Cys-Gly hydrolase activity. Given that Cys-Gly is a metabolite produced during the two-step catabolism of glutathione, and glutathione synthase was identified as an important enzyme for fitness under UVR, we investigated the enzymatic properties of SynPCC7942_1190. The recombinant protein was overexpressed in *E. coli*, and the purified 51-kDa protein migrated as a single band of the expected size on a sodium dodecyl sulfate‐polyacrylamide gel electrophoresis (SDS-PAGE) (gel (Fig. 6*A*). The substrate specificity of SynPCC7942_1190 was then assessed against Cys-Gly and L-leucine-p-nitroanilide (L-leu-p-NA) at pH 7.5. Using Cys-Gly as a substrate, a *K*_m_ of 1.21 mM was observed, whereas experiments with L-leu-p-NA yielded a *K*_m_ of 7.44 mM (Fig. 6*E*). No activity was detected against various other substrates including cystine (Cys-Cys), glycylglycine (Gly-Gly) or glutathione (Glu-Cys-Gly), suggesting specific and preferential Cys-Gly affinity for SynPCC492_1190 at pH 7.5 despite exhibiting leucyl aminopeptidase activity (Fig. 6*B*).

Given that the M17 family of proteases are metalloproteases, the purified protein was tested to determine metal requirements for Cys-Gly activity. In the presence of ethylenediaminetetraacetic acid (EDTA), no activity was observed (Fig. 6*C*). Maximal activity was observed with the addition of Mn^2+^, and a small degree of activity was observed with the addition of Co^2+^. No activity was observed with the addition of Ca^2+^, Zn^2+^, or Mg^2+^.

To assess the effect of pH on the activity of the enzyme, both Cys-Gly and L-leu-p-NA were tested as substrates across a pH gradient from 6.5 to 9.5 (Fig. 6*D*). Maximal enzymatic activity against Cys-Gly was observed at pH 7.5, while maximal activity against L-leu-p-NA occurred at pH 8.0. The enzyme was not as sensitive to pH changes against L-leu-p-NA when compared to Cys-Gly, similar to results published previously for an LAP from the pathogenic bacterium *Treponema denticola* (18).

### Synpcc7942_1190 Is Conserved across Ecologically Relevant Phytoplankton and Knockout Phenotype Is Conserved across Prokaryotic Species.

Proteins with strong sequence similarity to Synpcc7942_1190 were identified using BlastP (19) against the genomes of select ecologically relevant phytoplankton that are present in surface waters, such as *Fragilariopsis cylindrus*, *Prochlorococcus marinus*, *Pseudonitzschia multistriata*, *Symbiodinium* sp. CCMP2592, *Synechococcus* sp. WH7803, and *Trichodesmium erythraeum*. In each homologous protein, conservation of amino acids at the seven resides canonically involved in M17 family metal binding and catalysis were identified (*SI Appendix*, Fig. S1). Additionally, all 97 genomes from marine and brackish *Prochloroccus*, *Cyanobium*, and *Synechococcus* isolates available on the Cyanorak database v2.1 (20) encode a homologous LAP.

**Fig. 7.**
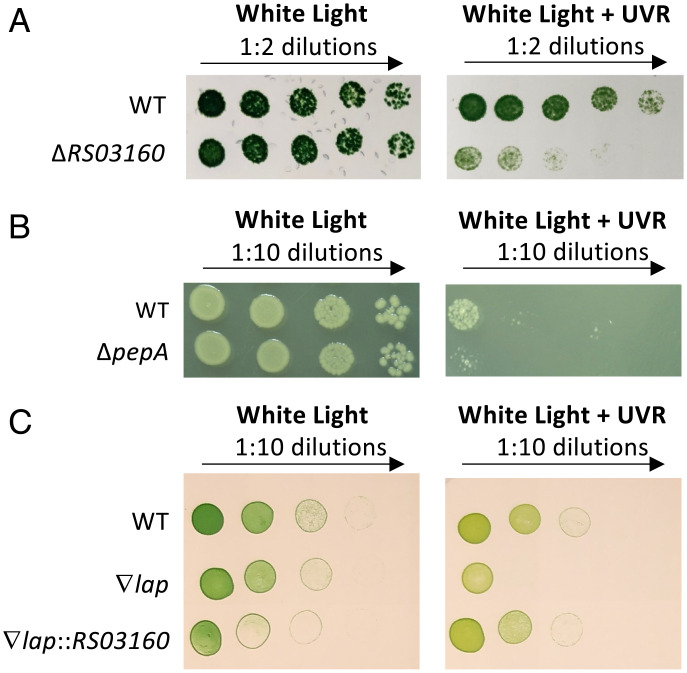
Spot tests reveal conservation of UV sensitivity in LAP-deficient mutants in a diversity of organisms. (*A*) The 5-µL spots of wild-type *Anabaena* sp. PCC 7120 and a markerless LAP mutant (Δ*RS03160*) grown under white light with or without UVR for 11 d. (*B*) The 5-µL spots of WT *E. coli* K-12 and Δ*pepA* grown under white light with or without UVR for 24 h. (*C*) The 5-µL spots of wild-type *S. elongatus,* the ∇*lap* mutant, and the ∇*lap* mutant complemented with an LAP from *Anabaena* sp. PCC 7120 (∇*lap*::*RS03150*), grown under white light with or without UV. The images shown are representative of those obtained in three independent experiments.

To test whether the UV-sensitive phenotype of the LAP-deficient mutant is observable among diverse species, deletion mutants of genes that encode the characteristic M17 family peptidase domains found in the *S. elongatus* LAP were generated in model organisms *Anabaena* sp. PCC 7120 (Δ*RS03160*) and *E. coli* (Δ*pepA*). The *E. coli* LAP gene chosen for this study was previously described to confer cysteinyl-glycinase activity in *E. coli* K-12 (21). Relative to the WT strain, the *Anabaena* sp. PCC 7120 Δ*RS03160* mutants produced fewer CFUs when grown under UVR in a 12-h light:12-h dark cycle relative to white light only, indicating conservation of the UV-sensitive phenotype across species of cyanobacteria (Fig. 7*A*). Similarly, the *E. coli* Δ*pepA* mutant resulted in reduced colony forming units when grown under UVR relative to white light only and the respective WT controls (Fig. 7*B*), indicating the conservation of the UVR-sensitivity phenotype of LAP knockouts broadly across prokaryotic genera. Additionally, to test whether the *lap* gene from *Anabaena* sp. PCC 7120 (*RS03160*) could be used to complement the ∇*lap* strain of *S. elongatus*, *RS03160* was cloned under the regulation of a P*trc* promoter and introduced into neutral site 1 of the chromosome in the ∇*lap* strain of *S. elongatus*. When spotted and grown on BG-11 agar, the WT phenotype was restored in the ∇*lap*::*RS03160* strain, corroborating conservation of the UVR tolerance mechanism in a leucyl aminopeptidase across cyanobacterial species (Fig. 7*C*).

## Discussion

This work has identified an unexpected protein, the aminopeptidase LAP, encoded by *Synpcc7942_1190*, as the strongest determinant of fitness under all UVR dosage conditions. This protein has a greater impact on UVR fitness than previously described proteins that strongly contribute to UVR tolerance through DNA repair, maintenance of the photosynthetic apparatus, and antioxidant synthesis (22, 23).

LAPs preferentially catalyze the hydrolysis of leucine residues at the N terminus of peptides and proteins and are often thought of as housekeeping enzymes. In addition to this functionality, LAPs have distinct and complex roles in diverse organisms ranging from bacteria to higher eukaryotes. For example, LAPs are involved in oxidative lens aging in the cattle species *Bos taurus* (24, 25), serve as a defense protein in the tomato plant *Lycopersicon esculentum* (26, 27), are a central component of H_2_S production, which may be important for pathogenesis in the pathogenic bacterium *T. denticola* (18), and aid the decay of particulate organic matter in marine systems (28). No LAPs have been previously implicated as a critical factor in UVR tolerance.

This study demonstrated that the *S. elongatus* LAP is capable of the hydrolysis of cysteinyl-glycine in addition to the expected activity against dipeptides containing leucine moieties. Several aminopeptidases from *E. coli* K-12, including PepA, PepB, PepD, and PepN, have been shown to confer cysteinyl-glycinase activity (21), and LAP cysteinyl-glycinase activity has also been reported in LAP purified from bovine lens, *Arabidopsis thaliana*, rat liver, and *T. denticola* (18, 25, 29, 30).

While the LAP demonstrated broad activity against l-Leu-p-NA across the pH gradient tested, there was no Cys-Gly hydrolase activity detected at pH levels at or above 8.5. Previous measurements of the cytosolic pH of *S. elongatus* have identified an increase in cytosolic pH from 7.3 ± 0.2 in the dark to 8.4 ± 0.1 in the light, due to H^+^ pumping from the cytosol into the thylakoid lumen triggered by photosynthetic activity (31). The results presented here suggest that, while the LAP may be functionally active as a leucyl aminopeptidase across all intracellular pH levels, it may preferentially function as a cysteinyl-glycinase when the cell is exposed to darkness. A similar phenomenon of pH dependence has been demonstrated for ribulose bisphosphate carboxylase/oxygenase (RuBisCO) and has been suggested for the regulation of chaperone function during light–dark cycles in *S. elongatus* (31, 32).

During the catabolism of glutathione (GSH), GSH is initially cleaved into glutamate and Cys-Gly by a gamma-glutamyltransferase (GGT), and Cys-Gly is subsequently degraded into glycine and cysteine by a cysteinyl-glycinase. Previous work in *Synechocystis* sp. PCC 6803, as well as *Emiliania huxleyi* and *Thalassiosira pseudonana*, have demonstrated that the onset of darkness triggers glutathione catabolism (33, 34). The affinity of the *S. elongatus* LAP toward cysteinyl-glycine under pH levels that are representative of the cytosolic pH during darkness likely facilitates the complete catabolism of glutathione during the dark period. The improved survival in UVR of the ∇*lap* mutant under constant light relative to a light–dark cycle provides further evidence this enzyme’s relevance to UVR tolerance is most pronounced during the dark period and reduced cytosolic pH levels.

There are several arguments for importance of a complete glutathione catabolism pathway in *S. elongatus*. Cysteine appears to be the main limiting amino acid for glutathione synthesis, in addition to being an important source of sulfur for other thiols and general cell metabolism in cyanobacteria (35–37). Additionally, it has been proposed that Cys-Gly has important redox properties and can be toxic in high quantities. This toxicity is likely due to the prooxidant nature of Cys-Gly, as it is a more reactive thiol than GSH, capable of starting iron redox-cycling processes that produce ROS and subsequent oxidative reactions (38). In the absence of a complete or efficient catabolism cycle, there is potential for the buildup of the dipeptide Cys-Gly within the cell, which results in a paucity of free cysteine and glycine. Abundant Cys-Gly would simultaneously create a prooxidant environment and a lack of Cys substrate to reallocate toward glutathione-dependent mechanisms of tolerance of oxidative stress conditions. It is possible that the severe fitness defect under UVR upon loss of this gene is due to the enzyme’s dual role as a housekeeping leucyl aminopeptidase during the day, and a cysteinyl-glycinase at night.

Previous screens of microorganisms have not implicated a cysteinyl-glycinase or leucyl aminopeptidase as being major factors in UVR tolerance (39, 40). The lack of differential expression of *lap* upon acute UVR exposure may explain this oversight in screens that have relied upon transcriptomic data (Fig. 2). Furthermore, the survival of *lap* insertional mutants under the constant light + UVR condition suggests that the strong UV-sensitive phenotype in *S. elongatus* would require subsequent exposure to a dark period to be detected. Thus, this phenotype may be overlooked in screens that focused on acute UVR exposure to photosynthetic organisms, or UVR exposure under constant light (39, 41).

Cyanobacteria such as *P. marinus* and marine *Synechococcus* spp., which dominate vast oceanic areas, and other ecologically relevant phytoplankton such as the diazotroph *T. erythraeum*, Bacillariophyceae such as *F. cylindrus* and the domoic acid producing diatom *P. multistriata*, and the dinoflagellate photosymbiont of corals *Symbiodinium microadriaticum,* all harbor LAPs with strong sequence similarity to Synpcc7942_1190 and identical amino acids at the seven residues canonically involved in M17 family metal binding and catalysis (*SI Appendix*, Fig. S1). This conservation of functional residues suggests that LAPs may support UVR stress tolerance in key primary producers residing in upper mixed layers of marine systems. The conservation of the UV-sensitive phenotype of LAP knockout strains in *E. coli* and *Anabaena* sp. PCC 7120 support the proposal that LAPs serve a UVR tolerance role in a diversity of organisms. It is possible that the *E. coli* LAP tested in this study may not be subject to the same pH dependent mechanism as the phototrophic *S. elongatus* LAPs yet is nevertheless important under UVR stress.

The only gene other than *Synpcc7942_1190* that confers moderate to strong fitness and has an obvious connection to the antioxidant cycling functional family is *gshB*, encoding glutathione synthase. GSH is canonically synthesized through two sequential adenosine triphosphate (ATP)-dependent steps, catalyzed by Glu-Cys ligase (*gshA*) and *gshB*; however, no homolog of *gshA* has been identified in *S. elongatus* PCC 7942. When GSH is oxidized (GSSG), it can be recycled back to GSH via glutathione reductase (*gor*; *Synpcc7942_0842*); however, mutation of *gor* had no more fitness cost under UVR stress than in white light alone. GSH is thought to be the major antioxidant present in cyanobacteria, playing a central role in ROS scavenging by providing electrons to various GSH-dependent enzymes (42). Δ*gshB* strains of *Synechocystis* sp. PCC 6803 have severe growth retardation in the presence of H_2_O_2_, as well as other forms of ROS, at concentrations that do not affect the WT or complemented strains, indicating that GSH is crucial for protection against diverse ROS species (33). Glutathione levels can be modulated by both light spectra and intensity in cyanobacteria (33, 43). Our results confirm the critical nature of GSH synthesis in *S. elongatus* under UVR and suggest that a complete catabolic cycle of GSH is necessary under natural conditions that involve UVR and a light–dark transition in *S. elongatus* as well as *Anabaena* sp. PCC 7120 and other microorganisms.

The two genes from the DNA repair functional family that strongly affect fitness under UVR are *phr*, encoding a deoxyribodipyrimidine type I photolyase, and *uvrC*, encoding excinuclease ABC subunit C. Deoxyribodipyrimidine photolyases catalyze the light-dependent repair of cyclobutyl pyrimidine dimers that form between adjacent DNA bases. Cyanobacteria from high-light biotopes tend to possess Phr, whereas those from low-light biotopes may not (22, 44). Phr is important for resistance against UVC radiation in *S. elongatus*; however, its enzymatic activity requires a light period following UVR exposure to drive catalysis, as would be experienced by an organisms in the natural environment (45). Our attempt to recreate a natural midday UVR exposure environment in the laboratory detected *phr* as critical for fitness. The UvrABC endonuclease enzyme complex proteins are encoded in all analyzed cyanobacterial genomes, supporting a core role in DNA repair (46). In *E. coli* UvrA initiates contact with damaged DNA, transfers the DNA to UvrB, and UvrC creates incisions around the damaged site. Subsequently DNA helicase II releases the incised nucleotide (47). Interestingly, in this study, *uvrC* was the only gene of the UvrABC complex to display a strong fitness effect (*uvrA* 0.15 ± 0.03 *P* < 0.05, *uvrB* −0.03 ± 0.08, *pcrA* −0.05 ± 0.23, under HUV). This result suggests redundancy and interchangeability of the functions of UvrA and UvrB.

The results of this screen suggest that the photosynthetic functional family genes *psb27*, *psb28-1*, and *psbJ* are fundamental for tolerating UVR stress in a cyanobacterium, likely by promoting the assembly and/or repair of PSII. Psb27 and Psb28-1 are needed for optimal growth under intermittent high-light/dark conditions, and Psb28-1 appears to be important for the synthesis of chlorophylls and apoproteins of chlorophyll-binding proteins (48–53). While two paralogs of Psb28 are present in *S. elongatus* (Psb28-1 and Psb28-2), *psb28-2* mutants conferred no observable fitness change under UVR stress in our screen, corroborating previous reports that Psb28-2 may serve an alternative function (54). PsbJ mutants have light-sensitive phenotypes due to improper assembly of PSII cores and deregulated electron flow, in addition to an accumulation of Psb27- and Psb28-containing PSII intermediates (53, 55, 56). Here, we identify these three photosystem genes as being of importance for UVR tolerance.

None of the genes identified as critical for fitness under UVR stress by the RB-TnSeq experiment, with the exception of *Synpcc7942_1511* (fitness estimate −0.5), was differentially expressed following an acute 15-min exposure to the HUV period (*SI Appendix*, Fig. S2). This highlights that studies focusing on differential expression at discrete timepoints may overlook many genes that are important for UVR tolerance. Three genes important for UVR fitness were down-regulated after 2 h of HUV exposure: *Psb28-1*, *Psb27*, and *Synpcc7942_1616*. This decrease in expression was observed in tandem with a global down-regulation of genes involved with photosystems I and II and phycobilin-containing light-harvesting complexes. This down-regulation by UVR may decrease photosynthetic activity, with subsequent up-regulation to be expected late in the dark period in anticipation of dawn. Notably, the hypothetical protein encoded by *Synpcc7942_1616* previously has been detected in PSI and PSII pulldown experiments, which when taken with the results of the RB-TnSeq experiment, suggests a potentially overlooked and important role of Synpcc7942_1616 in association with photosystem complexes (57).

The results of this study define the gene set that is critical for UVR tolerance under environmentally relevant UVR conditions in a model photosynthetic organism; however, it should be noted that in comparison to natural sunlight incident on Earth's surface, the spectral quality of the UV lamp used in this study is enhanced in the UVB relative to UVA, weighting the fitness effects observed toward the impact of short-wavelength UVR. It is likely that essential genes, such as superoxide dismutase, which are not assayed with the RB-TnSeq library, are also important for fitness under UVR stress, and that relevant genes with redundant homologs may have escaped detection. While the importance of such genes should not be overlooked, the utilization of an RB-TnSeq library in *S. elongatus* under realistic UVR conditions has corroborated the importance of several canonical gene functional families to UVR tolerance, such as DNA repair, photosystem maintenance, and glutathione synthesis, and highlighted the key role of a previously overlooked leucyl aminopeptidase with cysteinyl-glycinase activity, which may be conserved among many ecologically relevant phytoplankton.

## Materials and Methods

### Growth Conditions and Initial Growth Experiments.

*S. elongatus* PCC 7942, stored in our laboratory as AMC06, (*S. elongatus*) was grown to an initial OD_750_ of 0.6, and 50 mL of culture was inoculated in triplicate into quartz bioreactors (100 mL, outer diameter 30 mm, inner diameter 25 mm, length 200 mm). The bioreactors were suspended above white LED panels with diffusion screens at an incident intensity of 160 μmol photons·m^−2^·s^−1^ at the bottom of the bioreactors. Two Q-Lab UVA 340 lamps (QUV-UVA 340, Q Lab) were positioned perpendicular to the white LED panels. The spectral output of the Q-Lab UVA 340 lamp resembles the spectrum of sunlight incident on Earth’s surface from ∼290 to 360 nm, albeit lacking in relative intensity from ∼365 to 400 nm (Dataset S3). The tubes were positioned at various distances from the UV lamps, in triplicate, resulting in the following UVR conditions: high UVR (HUV, 1 mW·cm^−2^), medium UVR (MUV, 0.6 mW·cm^−2^), and low UVR (LUV, 0.4 mW·cm^−2^). UVR levels were measured with a Solarmeter Model 5.0 Standard UVA+B meter. A control group, PAR alone, was created by wrapping a single layer of Edmund Polyester High-Pass UV Filter Sheet (<10% transmission below 390 nm) around three bioreactors positioned at the same distance from the UVR source as the LUV condition. All bioreactors were bubbled with ambient air filtered through a 0.2-μm filter and subjected to a 12-h light:12-h dark regime, with activation of the UV lamp during the central 6 h of the light period to mimic highest UVR exposure during the midday solar zenith. OD_750_ was continually monitored, and cultures were diluted upon reaching an OD_750_ of ∼0.3 to maintain the optical thinness of the cultures, limiting the maximum attenuation of UVR across the 25-mm reactor to less than 30%.

### Growth Rates and Absorption Spectra.

*S. elongatus* was grown to an initial OD_750_ of 0.6, and 50 mL of culture was inoculated in triplicate into quartz bioreactors at an OD_750_ of 0.025. Growth rates were determined by measuring OD_750_ daily. Additionally, absorption spectra from 300 to 800 nm were recorded on a Tecan Infinite M200 plate reader, with BG-11 media used as a blank, and corrected to 0 at 750 nm. The spectra were normalized to the local maximum at 675 nm.

### ROS.

Samples were collected for analysis of ROS following the completion of three light:dark cycles. The fluorescent marker 2′,7′-dichlorodihydrofluorescein diacetate (H_2_DCFDA) was used following the methods described in Rastogi et al. (58). A total of 2.5 μL of 2 mM H_2_DCFDA was added to 1 mL of sample for a final concentration of 5 μM and incubated at 30 °C for 30 min. The fluorescence was then quantified at an excitation of 480 nm and an emission of 520 nm on a Tecan Infinite M200 plate reader with the gain manually set to 120. Fluorescence data were normalized to OD_750_ for each sample, and untreated-sample background fluorescence was then subtracted from treated-sample fluorescence values.

### RB-TnSeq Library Growth and Sampling.

A 1-mL aliquot of an *S. elongatus* PCC 7942 RB-TnSeq library, previously archived at −80 °C, was quickly thawed for 2 min at 37 °C, resuspended in two flasks of 100 mL BG-11 with kanamycin (Km), and incubated at 30 °C for 1 d at 30 μmol photons·m^−2^·s^−1^ without shaking (9). The culture flasks were then transferred to 70 μmol photons·m^−2^·s^−1^ on an orbital shaker. The cultures were subjected to a 12-h light:12-h dark regime for entrainment for 2 d, until the OD_750_ reached 0.25. The cultures were then combined and diluted to an OD_750_ of 0.025, and four replicates of 15 mL were spun down at 4,500 × *g* and frozen at −80 °C as time 0 samples to determine the population baseline. Aliquots of 50 mL of the RB-TnSeq outgrowth were then transferred to 12 quartz tubes and grown in identical conditions to the preliminary growth experiments for five generations. A total of 15 mL of culture from each tube was subsequently collected by centrifugation at 4,500 × *g*, and DNA was extracted from the pellet using a phenol-chloroform extraction (17).

### Barseq.

The method for Barseq analysis was conducted as previously described (59). In brief, amplification of barcodes from extracted DNA was conducted using 1 of 96 indexed forward primers for multiplexing, BarSeq_P2_ITXXX, and a common reverse primer, BarSeq_P1. PCR was performed in a final volumes of 50 µL using Q5 DNA polymerase, Q5 GC Enhancer (New England Biolabs), and the following thermocycler conditions: 1) 98 °C for 4 min; 2) 25 cycles of 30 s at 98 °C, 30 s at 55 °C, and 30 s at 72 °C; 3) and a final extension at 72 °C for 5 min. A total of 10 µL of each PCR product was then combined, purified with a DNA Clean and Concentrator Kit (Zymo Research), and quantified using a Nanodrop 2000 Spectrophotometer (Thermo Scientific). The pooled and purified product was then sequenced using Illumina HiSeq4000 SR75 by the IGM Genomics Center at the University of California San Diego.

### Fitness Calculations.

Fitness values were calculated using a previously described R script (10). In brief, the number of reads for each sample was used as a normalizing factor between samples. Any barcode falling outside or within the peripheral 20% of a gene coding sequence was ignored. Of the remaining barcodes, any genes represented by fewer than three barcodes were removed. For each barcode in each sample, a pseudocount of one was added to the number of reads, divided by the total number of reads for the sample, and the sample-normalized number of reads was log_2_ transformed. Any gene without at least 15 T0 reads was also removed. For each gene, maximum likelihood was used to fit a pair of nested linear mixed effects models to the sample- and read-normalized log_2_ transformed counts. Genes with significant fitness differences between the UVR condition and PAR only control were identified by comparing the difference in the −2·log likelihoods of the models to a χ^2^ distribution with one degree of freedom, estimating a *P* value, accounting for multiple testing by the FDR method of Benjamini–Hochberg (60), and selecting those genes with adjusted *P* values less than 0.02. We took the contract of *C_UVx_* − *C_PAR_* to be the estimated UV-specific fitness effect of knocking out a given gene. Mutants that had fitness effects below a confidence threshold (FDR adjusted *P* value <0.05) were not included. Mutants with gene disruptions conferring a fitness effect of ≥1 or ≤ −1 were classified as having a strong fitness effect, whereas mutants with gene disruptions conferring a fitness effect of ≥0.5 or ≤ −0.5 were classified as having a moderate fitness effect. Fitness scores of <0.5 or > −0.05 were classified as having negligible fitness effects.

### RNA-Seq Sampling and Library Preparation.

*S. elongatus* was grown under the HUV and PAR conditions described for the initial growth and RB-TnSeq experiments for 3 d prior to sampling 15 min and 2 h after the onset of the UVR period. Triplicate 10-mL samples at each timepoint were collected, and 2 mL of an ethanol-phenol stop solution was added on ice. Samples were centrifuged at 4,500 × *g* for 10 min at 4 °C, decanted, and the cell pellets were stored at −80 °C. Total RNA was isolated and purified using the Zymo Research Quick-RNA Fungal/Bacterial Microprep Kit from frozen cell pellets previously harvested according to the manufacturer’s protocols. Ribosomal RNA was removed from 1 μg total RNA with the use of a QIAseq FastSelect - 5S/16S/23S kit (Qiagen). The resulting rRNA-subtracted RNA preparations were made into libraries with the KAPA RNA HyperPrep kit incorporating short Y adapters and barcoded PCR primers. The libraries were quantified with a fluorescent assay (dsDNA AccuGreen quantitation kit, Biotium) and checked for proper size distribution and average size with a TapeStation (D1000 Tape, Agilent). Library pools were then assembled and a 1× SPRI bead cleanup was performed to remove traces of carryover PCR primers. The final library pool was quantified and run on an Illumina NovaSeq6000 with a PE50 run configuration. The sequencing depth ranged from 8.8 to 12.8 million reads per sample.

### RNA-Seq Differential Expression Analysis.

Differential expression analysis was conducted using the following R packages: Rsamtool (R package version 1.30.0), GenomeInfoDb (R package version 1.14.0.), GenomicFeatures (61), GenomicAlignments, GenomicRanges (61), and DESeq2 (62). The workflow was followed as described by Love et al. (62) and the differential expression was performed using DESeq2. Differentially expressed genes between the HUV and PAR condition were selected for an adjusted *P* value <0.05 (Benjamini–Hochberg correction for multiple testing) and an absolute log_2_ of fold change equal to or greater than 2.

### Construction of Mutants and Complemented Strains.

The plasmid for the insertional mutation of Synpcc7942_1190 (∇*lap*) was taken from the unigene set (UGS-24-F-1). The plasmid for complementation of the ∇*lap* mutant strain with the native *lap* gene and promoter (∇*lap*::*lap*) was created as follows: pAM5217, the destination vector for the gene for complementation, was generated via seamless assembly with a GeneArt Seamless Cloning and Assembly Kit (Thermo Fisher Scientific) of individual parts pCVD020 (NS1-Tc, pAM4836), pCVD002 (SpSm, pAM4818), and pCVD015 (ccdb-*Swa*I), available from the Cyanovector system (63). pAM5217 was then digested with *Swa*I (NEB), and a Gibson assembly (NEB) between the linearized product and the PCR product of the region 243 bp upstream to 71 bp downstream of SynPCC7942_1190 was performed, forming plasmid pAM5816 (Table 1). The SynPCC7942_1190 PCR product was generated using the primer pair 5217 to 1190-F and 5217 to 1190-R using standard protocols (*SI Appendix*, Table S1). The *lap* mutant strain was then transformed with the pAM5816 plasmid.

**Table 1.** List of plasmids used in this study

Plasmid name	Description	Source
UGS-24-F-1	Unigene set plasmid for insertional mutation of *Synpcc7942_1190*	(69)
pCVD002	SpR, SmR gene carried on a CYANO-VECTOR device (Sp, Sm, Ap)	(63)
pCVD015	Counter selectable *ccdB*-based cloning cassette carried on a CYANOVECTOR donor plasmid (Ap)	(63)
pCVD020	*S. elongatus* NS1 carried on a CYANO-VECTOR donor plasmid (Ap, Tc)	(63)
pAM2991	One-step cloning vector for overexpression with Ptrc promoter (Sp, Sm)	(64)
pAM5217	Cloning vector generated by seamless assembly of pCVD002, pCVD015, and pCVD020 (Sp, Sm, Tc)	This study
pAM5572	RSF1010-based broad host range plasmid for genome editing using Cpf1/CRISPR technology (Sp, Sm)	(11)
pAM5799	pAM5572 with gRNA spacer sequence targeting *alr0236*, and homology regions flanking *PCC7120DELTA_RS03160* (Sp, Sm)	This study
pAM5816	Plasmid for chromosomal integration of *Synpcc7942_1190* and native promoter (Sp, Sm)	This study
pAM5835	pAM2991 with PCR product of *PCC7120DELTA_RS03160* (Sp, Sm)	This study
pAM5836	*Synpcc7942_1190* cloned into a pET-28b(+) vector backbone with Strep-tag appended on 3′ end (*K*_m_)	This study

The plasmid for complementation of the *lap* mutant with the LAP derived from *Anabaena* sp. PCC 7120 (pAM5835) was created by linearizing pAM2991 (Addgene plasmid #40248) (64) with *Eco*RI and *Bam*HI, and performing a Gibson Assembly (NEB) between the linearized backbone and the PCR product of PCC7120DELTA_RS03160 (WP_010994414.1) using the primer pair 2991-A7120-LAP-F and 2991-A7120-LAP-R (*SI Appendix*, Table S1).

To generate the plasmid for the markerless deletion of a native LAP (PCC7120DELTA_RS03160) in *Anabaena* sp. PCC 7120, the oligos A7120_gRNA-LAP-F and A7120_gRNA-LAP-R were annealed to produce a guide RNA template and were cloned into the *Aar*I site of the CRISPR/cfp1 containing plasmid pAM5572 (11) by Golden Gate cloning (65). The guide RNA-containing plasmid was digested with *Kpn*I-HF (NEB) and dephosphorylated with Antarctic phosphatase, and the linearized backbone was gel purified. Homologous repair templates upstream and downstream of the target *PCC7120DELTA_RS03160* gene were generated by PCR with the primer pairs A7120-LAP-U-F/A7120-LAP-U-R and A7120-LAP-D-F/A7120-LAP-D-R and cloned into the linearized backbone using a three-part Gibson Assembly (NEB) (*SI Appendix*, Table S1). The final plasmid product (pAM5799) was verified by sequencing.

Transformation of *S. elongatus* was achieved using standard protocols (17). Genotyping of *S. elongatus* was performed using colony PCR with Taq DNA Polymerase (NEB). Transformation of *Anabaena* sp. PCC 7210 was achieved by biparental conjugation (66). Genotyping of *Anabaena* sp. PCC 7120 was performed using PCR with Q5 DNA Polymerase on DNA of isolate cultures extracted using phenol-chloroform (17).

### Recombinant Expression and Purification of SynPCC7942_1190 from *E. coli*.

Two primers (pet28-linear-streptag-F and pet28-linear-streptag-R; *SI Appendix*, Table S1) were used to linearize a commercially available pET-28b(+) vector (Millipore Sigma) with sequence to encode a Strep-tag on the N terminus of the protein product. An additional primer pair (pet28-1190-streptag-F and pet28-1190-streptag-R, *SI Appendix*, Table S1) was used to amplify *Synpcc7942_1190* with appropriate overhangs for subsequent Gibson Assembly (NEB). The resulting plasmid (pAM5836) was used to transform *E. coli* DH5α cells for plasmid generation and sequence confirmation. The plasmid was subsequently moved into *E. coli* BL21 (DE3).

Plasmid-bearing *E. coli* BL21 (DE3) strains were grown to OD_600_ of 0.5 with 50 mg/mL kanamycin before overnight induction of protein expression at room temperature by the addition of 200 µM isopropyl β-D-1-thiogalactopyranoside. Cells were then collected by centrifugation and the pellet was resuspended in 50 mL of StrepTactin wash buffer (50 mM Tris⋅HCl, pH 8.0, 150 mM NaCl, 5% glycerol). The resuspended cells were disrupted on ice by French press, and the lysate was clarified by centrifugation at 27,000 × *g* (Sorvall SS-34 rotor) at 4 °C for 30 min. Recombinant protein was purified using a StrepTactin XT Superflow resin column (IBA Lifesciences). The column was washed with 20× the column volume of StrepTactin wash buffer, followed by protein elution with same buffer containing 50 mM biotin, and collected in six elution aliquots. The elution aliquots were run on SDS-PAGE to assess purity, and the central three elution aliquots were pooled.

### Enzymatic Assays.

The standard reaction buffer for all cysteinyl-glycinase assays was 50 mM Tris⋅HCl (pH 7.5 or 8.0), 2 mM Cys-Gly, and 0.2 mM MnCl_2_, unless another pH, substrate concentration, or cation is indicated. The purified LAP protein was incubated at 5 μg/mL for 20 min at 37 °C and terminated with 5% trichloroacetic acid (TCA). The reaction product L-cysteine was measured as described by Gaitonde (67). L-cysteine concentrations were determined by absorbance at 560 nm, after subtracting a blank.

Leucine aminopeptidase activity was determined using L-Leu-p-NA (Millipore Sigma) as a substrate (68). The standard reaction buffer for these reactions was 50 mM Tris⋅HCl (pH 7.5 or 8.0), 2 mM L-Leu-p-NA, and 0.2 mM MnCl_2_, unless another pH, substrate concentration, or cation is indicated. The purified LAP protein was incubated at 5 μg/mL for 20 min at 37 °C and terminated with 5% TCA. The concentration of the reaction product was determined by absorbance at 405 nm, after subtracting a blank. All assays were performed in triplicate.

### LAP Sequence Alignment.

Proteins with sequence similarity to Synpcc7942_1190 were identified using Blastp (19) against the genomes of *Anabaena* sp. PCC 7120 (accession WP_010994414.1), *F. cylindrus* (accession OEU09452.1), *P. marinus* (accession WP_158467072.1), *P. multistriata* (accession VEU35739.1), *Symbiodinium* sp. CCMP2592 (accession CAE7247217.1), *Synechococcus* sp. WH7803 (accession A5GN62.1), and *T. erythraeum* (accession Q11A96.1). A multiple alignment of the amino acid sequences was conducted with Geneious Alignment in Geneious Prime version 2022.0.2. Additionally, Synpcc7942_1190 was queried against the Cyanorak database v2.1 (20).

## Supplementary Material

Supplementary File

Supplementary File

Supplementary File

Supplementary File

## Data Availability

RNA-Seq and RB-TnSeq data generated in this study have been deposited in the NCBI Sequence Reads Archive (SRA) database, https://www.ncbi.nlm.nih.gov/sra (Bio-Project identifier PRJNA854269) (70).
