# Supplementary material for: An unexpected role for leucyl aminopeptidase in UV tolerance revealed by a genome-wide fitness assessment in a model cyanobacterium
Source: Proc Natl Acad Sci U S A. 2022 Nov 2;119(45):e2211789119. doi: 10.1073/pnas.2211789119 (PMC9659335; doi:10.1073/pnas.2211789119)
Supplement: Supplementary File [file pnas.2211789119.sapp.pdf]

PNAS

Supporting Information for

An Unexpected Role for Leucyl Aminopeptidase in UV Tolerance Revealed by a Genome-wide Fitness Assessment in a Model Cyanobacterium

Elliot L. Weiss, Mingxu Fang, Arnaud Taton, Richard Szubin, Bernhard Ø. Palsson, B. Greg Mitchell, Susan S. Golden

Susan S. Golden
Email: sgolden@ucsd.edu

This PDF file includes:

Figures S1 to S2
Table S1
Legends for Datasets S1 to S3

Other supporting materials for this manuscript include the following:

Datasets S1 to S3

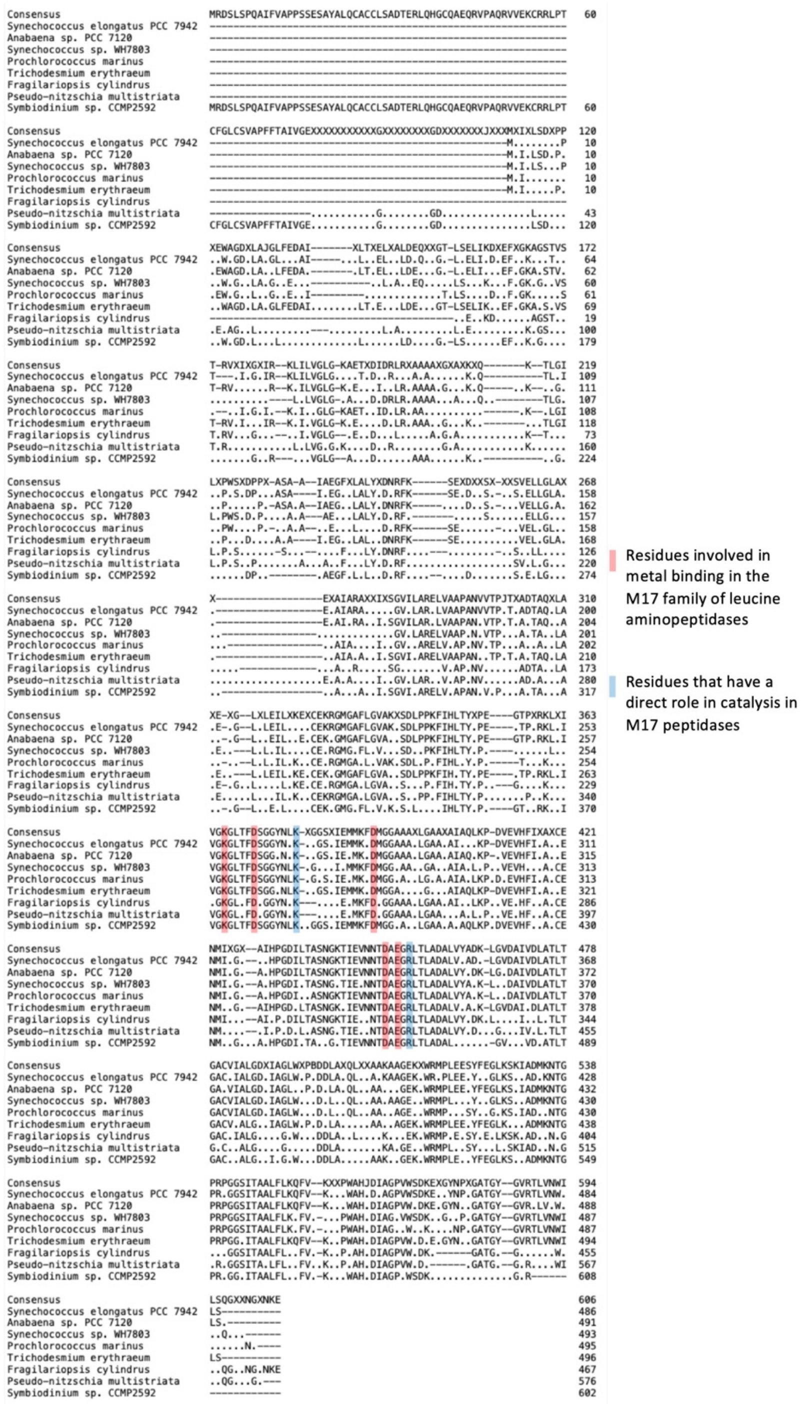

Fig. S1. Alignment of the *S. elongatus* LAP with BlastP matches from environmentally relevant phytoplankton, illustrating the conservation of amino acids at the seven residues canonically involved in M17 family metal binding and catalysis. Red column highlights indicate residues involved in metal binding in the M17 family of leucine aminopeptidases, and blue column highlights indicate residues that have a direct role in catalysis in M17 peptidases.

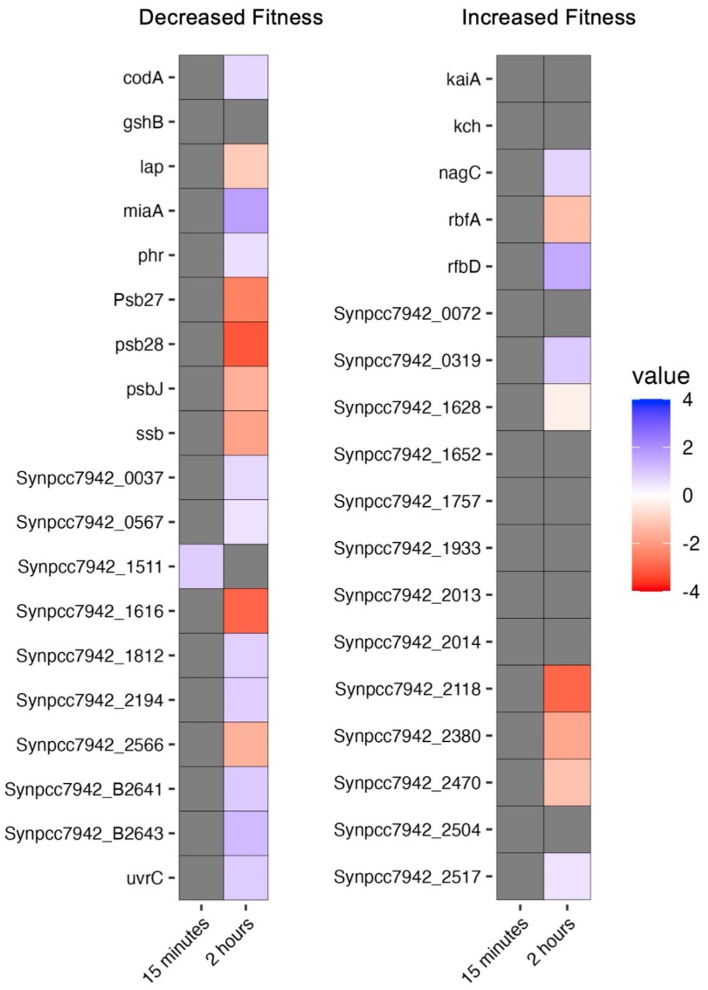

Fig. S2. Differential expression after 15 min or 2 h of HUV exposure of genes identified as conferring a strong increase or decrease in fitness when disrupted.

Table S1. List of primers used in this study.

**Primer Name**	**Sequence 5' → 3'**
5217-1190-F	ATAACCCAGGGATTTCAAGATCGCTTTCAAGAGCGTGC
5217-1190-R	GGAGCTCCTTCATTTCAGTAGAGTCTAGCTTGATCATCAG
2991-A7120-LAP-F	GATAACAATTTCACACAGGAAACAGACCATGGCAATTCAACTGAGTGATAAGC
2991-A7120-LAP-R	GCTTGCATGCCTGCAGGTCGACTCTAGAGCTACTCACTCAATACCCAATCTAC
pet28-linear-streptag-F	AGCTCCGTCGACAAGCTTG
pet28-linear-streptag-R	CTGCGGGTGGCTCCATTCCATGGTATATCTCCTTCTTAAAGTTAAAC
pet28-1190-streptag-F	CATGGAATGGAGCCACCCGCAGTTCGAAAAGTCGGCCACATTTCAGGCGATCGCCAC
pet28-1190-streptag-R	GCAAGCTTGTCGACGGAGCTCTAGGACAGCACCCAGTTCA
A7120_gRNA-LAP-F	AGATCAAACCAGAAGGCACACCCA
A7120_gRNA-LAP-R	AGACTGGGTGTGCCTTCTGGTTTG
A7120-LAP-U-F	TGCGGTAGTTGGTACCAAGTATCCCCAGGAATCACCA
A7120-LAP-U-R	CCTACCCTGCTTTCCTGTACCAGTATTGCTC
A7120-LAP-D-F	GGAAAGCAGGGTAGGGGTATAGGGGTGTAG
A7120-LAP-D-R	CCCCCGATGTCGACGGTACCGGAGGATTTGCAGAATTACACC

Dataset S1 (separate file). Differential expression of genes after 15 min or 2 h of HUV exposure. Essentiality status of all genes included in file (1)**.**

.

Dataset S2 (separate file). RB-TnSeq fitness estimates for HUV, MUV, and LUV conditions

Dataset S3 (separate file). Spectral power distribution of UVA-340 Fluorescent Lamp as provided by the manufacturer (2).

SI References

1. B. E. Rubin, *et al.*, The essential gene set of a photosynthetic organism. *Proc. Natl. Acad. Sci.* **112**, E6634-E6643 (2015).
2. Q-Lab, Technical Bulletin LU-8052 - Spectral Power Distribution for QUV® with UVA-340 Fluorescent Lamps (2011).
